# Long-term follow-up of colorectal cancer screening attendees identifies differences in *Phascolarctobacterium* spp. using 16S rRNA and metagenome sequencing

**DOI:** 10.3389/fonc.2023.1183039

**Published:** 2023-04-27

**Authors:** Cecilie Bucher-Johannessen, Einar Elvbakken Birkeland, Elina Vinberg, Vahid Bemanian, Geir Hoff, Paula Berstad, Trine B. Rounge

**Affiliations:** ^1^ Department of Tumor Biology, Institute of Cancer Research, Oslo University Hospital, Oslo, Norway; ^2^ Department of Research, Cancer Registry of Norway, Oslo, Norway; ^3^ Centre for Bioinformatics, Department of Informatics, University of Oslo, Oslo, Norway; ^4^ Department of Pathology, Akershus University Hospital, Oslo, Norway; ^5^ Department of Research, Telemark Hospital Skien, Skien, Norway; ^6^ Section for Colorectal Cancer Screening, Cancer Registry of Norway, Oslo University Hospital, Oslo, Norway; ^7^ Centre for Bioinformatics, Department of Pharmacy, University of Oslo, Oslo, Norway

**Keywords:** archived fecal samples, colorectal cancer screening, microbiome, 16S rRNA, sequencing, metagenome, long term follow-up

## Abstract

**Background:**

The microbiome has been implicated in the initiation and progression of colorectal cancer (CRC) in cross-sectional studies. However, there is a lack of studies using prospectively collected samples.

**Methods:**

From the Norwegian Colorectal Cancer Prevention (NORCCAP) trial, we analyzed 144 archived fecal samples from participants who were diagnosed with CRC or high-risk adenoma (HRA) at screening and from participants who remained cancer-free during 17 years of follow-up. We performed 16S rRNA sequencing of all the samples and metagenome sequencing on a subset of 47 samples. Differences in taxonomy and gene content between outcome groups were assessed for alpha and beta diversity and differential abundance.

**Results:**

Diversity and composition analyses showed no significant differences between CRC, HRA, and healthy controls. *Phascolarctobacterium succinatutens* was more abundant in CRC compared with healthy controls in both the 16S and metagenome data. The abundance of *Bifidobacterium* and *Lachnospiraceae* spp. was associated with time to CRC diagnosis.

**Conclusion:**

Using a longitudinal study design, we identified three taxa as being potentially associated with CRC. These should be the focus of further studies of microbial changes occurring prior to CRC diagnosis.

## 1 Introduction

Colorectal cancer (CRC) is the third most common cancer in men and the second in women worldwide (1, 2). Symptoms are often unspecific, and many cases are detected at an advanced stage with reduced prospects for curative treatment. The progression toward CRC passes through stages of molecular and morphological changes from small and benign through advanced adenoma and finally to CRC. This adenoma–carcinoma sequence is estimated to take on average between 10 and 15 years (3). This time window provides an opportunity to screen and potentially remove lesions that have not yet developed into clinical cancer and advanced stages (3, 4). Several randomized studies have estimated that CRC screening by fecal tests reduces CRC mortality by 15%-30% (5–8). However, fecal-based tests are hampered by both poor sensitivity and specificity, particularly for the detection of CRC precursor lesions (9). Therefore, there is a need for additional markers that can be used in fecal-based screening for CRC precursor lesions.

Analyses of the gut microbiome composition, diversity, and functional potential have demonstrated that the gut microbiome of CRC patients is different from that of their healthy counterparts, making it a source of potential biomarkers for CRC (10–14). The presence of certain microbes is strongly associated with CRC. The most frequently reported are *Fusobacterium nucleatum*, *Bacteroides fragilis*, and pks^+^ *Escherichia coli*. The proposed mechanisms for the role of the microbiome in carcinogenesis include DNA damage through the secretion of genotoxic compounds, the induction of inflammation, and the activation of procarcinogenic signaling pathways (15, 16). While it has been shown that fecal tests in combination with microbial biomarkers are superior at separating healthy controls from CRC to that of a fecal test alone (17, 18), no specific bacterial profile is recognized as a biomarker for CRC. Still, less is known about the role of the microbiome in the early stages of carcinogenesis.

To identify a precancerous signal in the microbiome, there is a need for studies with sample collection prior to diagnosis and long-term follow-up. We performed microbiome sequencing on archived stool samples collected from screening attendees from the Norwegian Colorectal Cancer Prevention (NORCCAP) trial, with a 17-year follow-up time after sigmoidoscopy screening. This study included both screening-detected cancers and CRC precursor lesions, as well as incident post-screening cancers, and healthy controls. We aimed at detecting community-wide and specific differences in the microbial profiles between CRC, high-risk adenoma (HRA), and healthy controls.

## 2 Material and methods

### 2.1 Study design and participants

**Figure 1:**
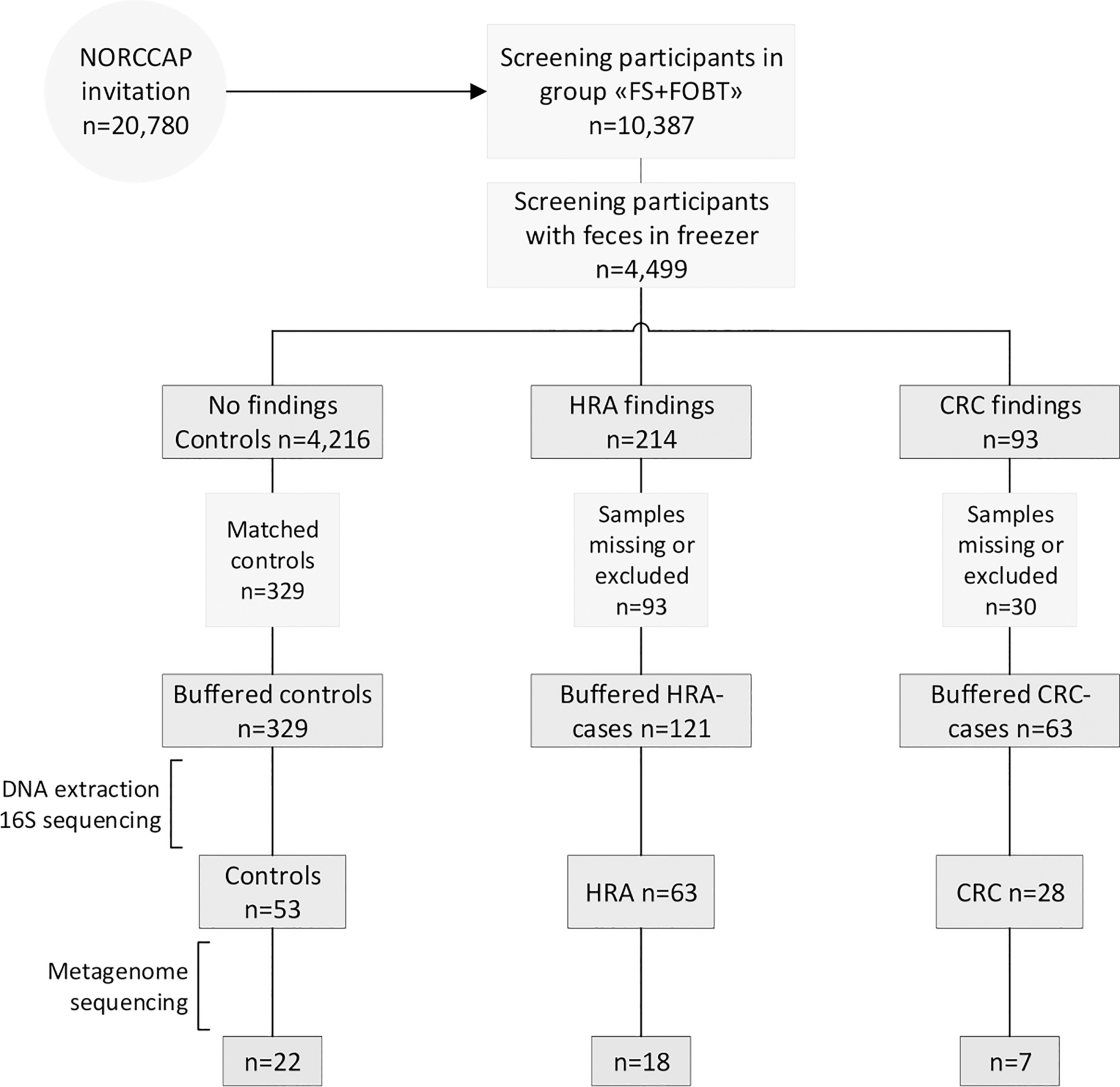
Recruitment flowchart. Half of the NORCCAP participants were invited to deliver a stool sample in addition to participating in sigmoidoscopy screening. Half of these fecal samples were stored below −20°C. A subset of samples diagnosed with CRC and HRA and healthy controls were included in the study and homogenized in preservation buffers. Those with sufficient DNA extracted were included in 16S rRNA (*n* = 144) and metagenome sequencing (*n* = 47). FS, flexible sigmoidoscopy; FOBT, fecal occult blood test.

Details of the NORCCAP trial have been described previously (19–21). Briefly, NORCCAP was a randomized clinical trial in which 20,780 individuals were offered sigmoidoscopy screening in the intervention arm, and it was performed in 1999-2000 (age group 55-64) and 2001 (age group 50-54). The study recruited participants directly from the population registry of the Norwegian counties Oslo and Telemark. All participants were examined with flexible sigmoidoscopy, while 10,387 participants additionally delivered stool samples for an immunochemical fecal occult blood test (iFOBT – FlexSure OBT) and a fresh-frozen stool sample for biobanking. We selected a subset of participants with archived fresh-frozen fecal samples for microbiome analyses (**Figure 1**). The participants’ full CRC history was retrieved from the Cancer Registry of Norway in 2015 by using personal identification numbers and included the ICD-10 coded diagnoses C18, C19, and C20. We included all participants diagnosed with CRC at screening or by registry follow-up. Individuals with high-risk adenomas were defined as those presenting with one or more adenomas of ≥10 mm, with high-grade dysplasia or villous components regardless of polyp size, or those with three or more adenomas regardless of size, dysplasia, and villosity. We included a subset of HRA samples matching the CRC group on sex, age, and examination date. The control group was selected from a pool of participants with no findings (i.e., no lesions) at the screening examination (including low-risk adenomas) and who remained cancer-free during follow-up. Controls were selected by matching sex, age, and examination date to the CRC and HRA groups. Samples that were missing from the freezer, had a low amount of stool, or had no DNA extracted (none detected by Qubit) were excluded. All methods were carried out in accordance with the Declaration of Helsinki. All participants signed the informed consent that their samples and data could be used for research upon enrolment in the study. The study and all experimental protocols received ethical approval from the Regional Committees for Medical and Health Research Ethics in South-Eastern Norway (ref: 22337).

### 2.2 DNA extraction, library preparation, and sequencing

Participants were asked to collect stool samples immediately after defecation at home in 20-ml vials and to store the samples for at most 7 days in a freezer (−20°C) before sigmoidoscopy screening. The samples were delivered to either of the two screening centers in Oslo or Telemark at the time of sigmoidoscopy screening where further storage was at −20°C. We have previously demonstrated the feasibility of obtaining microbiota profiles from these archived stool samples (22). Prior to DNA extraction, the samples were thawed, homogenized, and mixed with OMNIgene gut buffer. The stool samples in NORCCAP have not been subjected to freeze–thaw cycles previously, with a few exceptions that have been thawed once only. The extraction of DNA was carried out using the QIAsymphony automated extraction system, using a QIAsymphony DSP Virus/Pathogen Midi Kit (Qiagen, Hilden, Germany), after an off-board lysis protocol with some modifications. Each sample was lysed with bead beating: a 500-µl sample aliquot was transferred to a Lysing Matrix E tube (Solon, USA:MP Biomedicals) and mixed with 700 µl of phosphate-buffered saline (PBS). The mixture was then shaken at 6.5 m/s for 45 s. After the bead beating, 800 µl of the sample was mixed with 1,055 µl of “off-board lysis buffer” (proteinase K, ATL buffer, ACL buffer, and nuclease-free water) and incubated at 68°C for 15 min for lysis. Nucleic acid purification was performed on the QIAsymphony extraction robot using the Complex800_OBL_CR22796_ID 3489 protocol. Purified DNA was eluted in 60 µl of AVE buffer (Qiagen, Hilden, Germany). DNA purity was assessed using a NanoDrop 2000 spectrophotometer (Thermo Fisher Scientific, MA, USA), and the concentration was measured using a Qubit instrument (Thermo Fisher Scientific, MA, USA).

After DNA extraction and sample quality assessment, the libraries were prepared for 16S rRNA and shotgun metagenome sequencing. In total, 144 samples had sufficient DNA for 16S rRNA sequencing. Sample amplification was carried out using 16S primers S-D-Bact-0341-b-S-17 (5′CCTACGGGNGGCWGCAG′3) and S-D-Bact-0785-a-A-21 (5′GACTACHVGGGTATCTAATCC′3) to amplify the V3-V4 regions (23). Amplification was performed using the TruSeq (TS)-tailed 1-step amplification protocol (24) with random spacers to shift the sequencing start. Paired-end 300 bp sequencing of PCR amplicons was performed on the Illumina MiSeq instrument (Illumina, Inc., CA, USA) (**Figure S1A**). Forty-seven of the samples had sufficient DNA for additional whole-genome shotgun sequencing (**Figure S1B**). The metagenomes provide additional taxonomical resolution and improved estimates of functional potential and were used for validation of the 16S rRNA sequencing results. Samples were cleaned up and concentrated using AMPure XP (Beckman Coulter, IN, USA) and normalized to a total input of 4 ng of dsDNA. Sequencing libraries were prepared using the Riptide protocol (Twist Bioscience HQ, CA, USA) and sequenced on Illumina NovaSeq paired-end 2 × 130 bp. The Riptide protocol includes linear amplification with random primers and dideoxy nucleotide-induced self-termination, thereby avoiding DNA fragmentation (25). Sequencing was performed at FIMM Technology Centre in Helsinki, Finland.

### 2.3 Bioinformatics analyses

Initial quality control of 16S sequencing reads included the removal of short reads (<50 bp) and low-quality bases with average quality across four bases below 30 using Trimmomatic v0.35.2 (26). The removal of primer sequences was performed using Cutadapt v2020.2.0 (27) with the following options: forward primer: CCTACGGGNGGCWGCAG, reverse primer: GACTACHVGGGTATCTAATCC, primer error 0.1, and primer overlap 3. Fastqc and multiqc (28) analyses were performed before and after trimming to ensure high-quality data. Reads were imported into Qiime2 v2020.2.0 (29), and amplicon sequence variant (ASV) classification was performed using the Divisive Amplicon Denoising Algorithm 2 (DADA2) plugin (30), including length trimming, merging, denoising, and chimera removal. ASV classification was carried out using the SILVA 16S rRNA database v132 (31) at a 97% similarity threshold. ASV data were filtered for the mitochondria and chloroplasts and were rarefied to a depth of 9,000 reads for each sample. Metagenome functional profiles were predicted from the 16S data using Phylogenetic Investigation of Communities by Reconstruction of Unobserved States 2 (PICRUSt2) v2.3.0 (32) with default settings, using rarefied count tables as input and mapping to the MetaCyc database giving pathway abundance.

Metagenome reads were trimmed using Trimmomatic v0.66.0 (26) with a sliding window approach in which reads with average quality across four bases below 30 or a read length of less than 30 base pairs were discarded. Following trimming, Bowtie2 v2.4.2 (33) and Samtools v1.12 (34) were used with default settings to remove reads mapping to the human genome. MetaPhlAn3 v3.0.4 (35) was used for taxonomic classification with default parameters. Percent abundances generated by MetaPhlAn3 were transformed into count-like tables by multiplying by the number of quality-trimmed reads per sample and dividing by 100. HUMAnN3 v3.0.0.alpha.2 was used to profile gene families encoding microbial pathways, aggregating the data according to MetaCyc annotations using the UniRef90 v201901 database (35). Pathway abundance data were corrected for sequencing depth by dividing by the number of trimmed reads and multiplying by 10^6^.

### 2.4 Statistical analysis

All statistical analyses were performed using R v3.5.3 and visualized using ggplot2 v3.3.2 (36). To assess the differences between CRC, HRA, and the control group, statistical tests were made contrasting all three groups or by combining CRC and HRA. Additionally, analyses were performed within the CRC group, using time to diagnosis as the dependent variable. Differences between the three groups were evaluated using the chi-square test for comparisons of two categorical variables and the Kruskal–Wallis test (or the Mann–Whitney *U* test for two-group comparisons) or Spearman`s correlation for comparisons of a continuous variable with a categorical and continuous variable, respectively. Statistical associations were considered significant at the *p <* 0.05 level.

Microbial diversity was measured on ASV and species level for 16S and metagenome data, respectively. Alpha diversity was determined using richness, Shannon, and inverse Simpson indices. Beta diversity was calculated using Bray–Curtis dissimilarity, as implemented in the Phyloseq R package v1.26.1 (37). Associations between beta diversity and CRC, HRA, and healthy controls were evaluated using permutational analysis of variance (PERMANOVA) with 999 permutations after adjustment for the participant’s sex and screening center, as implemented in the adonis function of the R package vegan v2.5-7 (38).

Differential abundance analyses were performed independently on ASV/species, genus, phylum, and pathways and were adjusted for sex and screening center. Before differential abundance analyses, we applied low abundance filtering, retaining all taxa/pathways with a read count of at least 10 in at least 10% of samples. Differential abundance analyses were performed using a negative binominal model-based Wald test implemented in the DESeq2 package v1.22.2 (39) with the type (poscounts) to account for the sparsity of microbiome data, and *p*-values were false discovery rate (FDR)-adjusted to control for multiple testing.

## 3 Results

### 3.1 Study population

**Figure 2:**
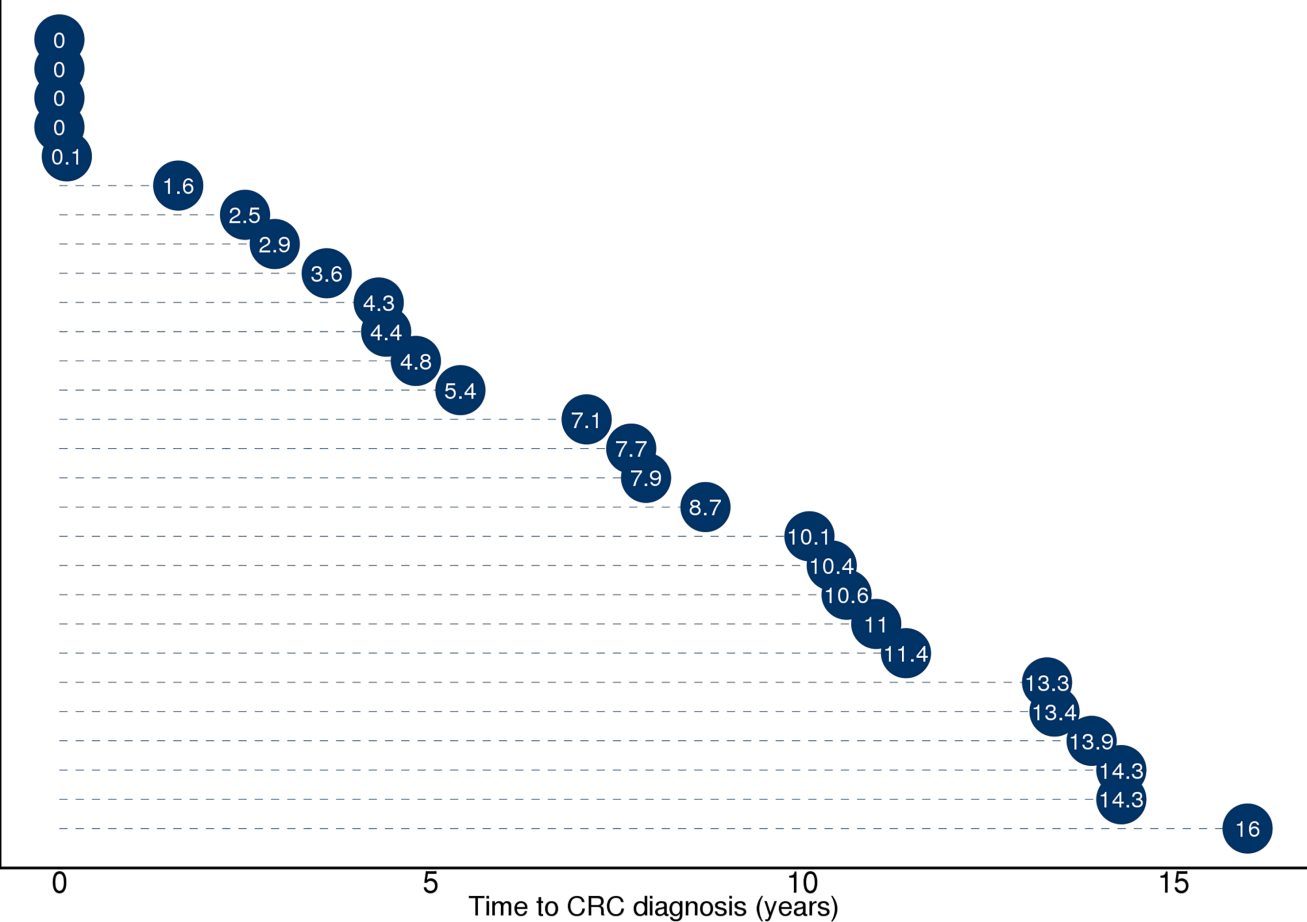
Illustration of time from sample collection to diagnosis (in years) for the 28 participants who received a CRC diagnosis during screening or 17 years of follow-up. Full diagnosis and cancer history was retrieved from the Cancer Registry of Norway and included the ICD-10 coded diagnosis C18, C19, and C20. Five participants with time to diagnosis ≤1 year received the diagnosis during screening. Twenty-three study participants received a CRC diagnosis during follow-up ≥1 year.

Stool samples from 144 NORCCAP screening participants were selected for 16S sequencing based on registry follow-up data and initial screening results. Metagenome sequencing was also performed on 47 of these with the highest DNA amounts. All 144 participants in this study underwent sigmoidoscopy. Five cases of CRC (3.5%) were detected during screening. Based on registry follow-up, 23 (16%) participants received a CRC diagnosis within 17 years after screening (**Figure 2** and **Table 1**). The median time from screening to CRC diagnosis was 7.4 years (range 0-16 years), and the median age at CRC diagnosis was 65.7 years (range 54-77), including both screening-detected and follow-up diagnosed CRC. Other screening-detected lesions included 63 HRAs (44% of the study participants). Fifty-three (37%) participants had no findings of adenomas or CRC during sigmoidoscopy and were cancer-free during follow-up; these constituted the control group. The median age for all groups at the sample collection was 57 years (range 51-65). We observed a significantly different distribution of sex and screening center between CRC, HRA, and healthy controls (*p* < 0.05). In total, 87 (60%) samples were from male participants and 89 (62%) samples were from the Telemark screening center.

**Table 1:** Characteristics of the study participants and samples.

	16S (*n* = 144)			Metagenome (*n* = 47)		
	Control	HRA	CRC	Control	HRA	CRC
Men (%)	36 (25)	41 (28.5)	10 (6.9)	15 (31.9)	12 (25.5)	2 (4.3)
Women (%)	17 (11.8)	22 (15.3)	18 (12.5)	7 (14.9)	6 (12.7)	5 (10.6)
Telemark (%)	39 (27)	37 (25.7)	13 (9)	16 (34)	11 (23.4)	5 (10.6)
Oslo (%)	14 (9.7)	26 (18)	15 (10.5)	6 (12.8)	7 (14.9)	2 (4.3)
Age at sampling, median (range)	57 (51-64)	57 (51-64)	60.5 (51-65)	57 (54-64)	58 (53-64)	61 (55-65)
Age at diagnosis, median (range)	–	–	65.7 (54-77)	–	–	65.8 (61.1-74.3)
Time to diagnosis, median (range)	–	–	7.4 (0-16)	–	–	4.8 (0-14)
Quality trimmed reads, median (range)	61,184 (10,261-416,286)	48,014 (5,163-493,315)	48,314 (23,701-510,589)	7,356,487 (757,832-16,480,674)	5,146,988 (998,978-20,095,193)	7,248,142 (1,757,061-15,370,089)
Excluded	0	2^a^ ^b^	0	0	1^b^	0

a One individual was excluded from 16S diversity analyses due to the rarefaction criterion of at least 9,000 reads.

b One individual was excluded from all the analyses (in both the 16S and metagenome datasets) because of an Escherichia coli infection.

### 3.2 Gut microbiome diversity

16S sequencing of 144 samples generated 11.8 million trimmed reads with a median read depth per sample of 50,205 (range 5,163-510,589). We identified a total of 7,228 ASVs mapped to 337 species, 229 genera, and 18 phyla. The median number of observed ASVs was 213.5 (range 79-603). Metagenomic sequencing of 47 samples resulted in 361 million trimmed reads with a median read depth of 6.2 million reads (0.76-20.1). In total, 561 taxa were identified, including 323 species, 116 genera, and 8 phyla. The median number of species per sample was 73 (34–107). ASV distribution for individual samples showed one sample with 83% of reads belonging to two ASVs within the genus *Escherichia-Shigella*. This was confirmed in the metagenome data in which 95% of reads belonged to the species *E. coli*. As this indicated an unrelated acute infection, the sample was excluded from further analyses (**Figure S2**).

**Figure 3:**
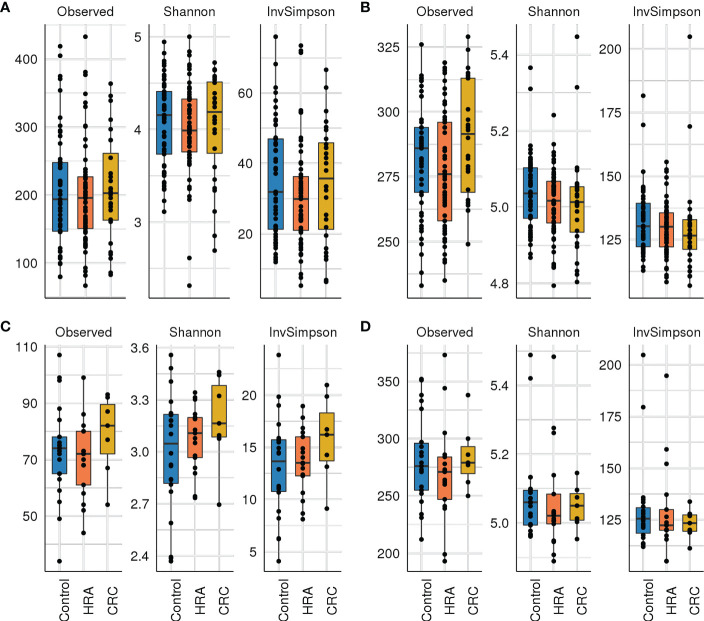
Alpha diversity: box plots with taxa/pathway richness (observed), Shannon, and inverse Simpson (InvSimpson) diversity indices in CRC, HRA, and controls for **(A)** amplicon sequence variants from 16S sequencing data, **(B)** estimated MetaCyc pathways derived from 16S data, **(C)** species abundance based on metagenome shotgun sequencing, and **(D)** MetaCyc pathways based on metagenome shotgun sequencing. No significant (*p* > 0.05) associations were identified.

**Figure 4:**
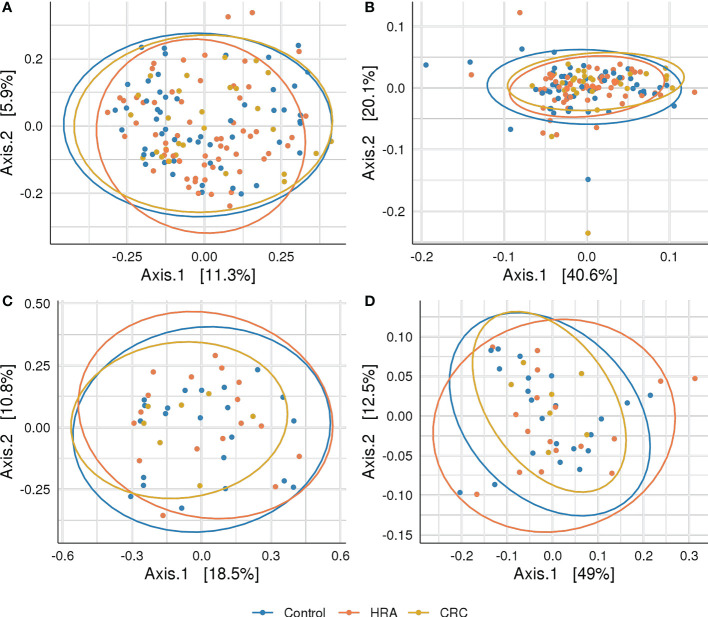
Beta diversity: PCoA plots with Bray–Curtis dissimilarity indices between CRC, HRA, and controls for **(A)** amplicon sequence variants from 16S sequencing data, **(B)** MetaCyc pathways derived from 16S sequencing data, **(C)** species abundance based on metagenome shotgun sequencing, and **(D)** MetaCyc pathways based on metagenome shotgun sequencing. Ellipses describe 95% of group variation for the principal coordinate axes. No significant (*p* > 0.05) associations were identified.

Rarefying 16S data to 9,000 reads resulted in the exclusion of one sample with lower sequencing coverage, leaving 142 samples for 16S diversity analyses. Forty-six samples were used for metagenome diversity analyses. We found no significant differences in alpha (unadjusted) or beta diversity of taxa or pathways between CRC, HRA, and healthy controls (**Figures 3A–D**, **4A–D**, *p* > 0.05 for all comparisons). This finding remained consistent when grouping CRC and HRA cases together, when looking at the time to diagnosis, when considering the metagenome data, and when adjusting for sex and screening center.

### 3.3 Differentially abundant taxa and pathways

We evaluated the differences in the abundance of ASV/species, genus, phylum, and pathways between the outcome groups. We further assessed the associations of ASVs with the time elapsed from sample collection to CRC diagnosis.

#### 3.3.1 CRC *vs*. control

**Figure 5:**
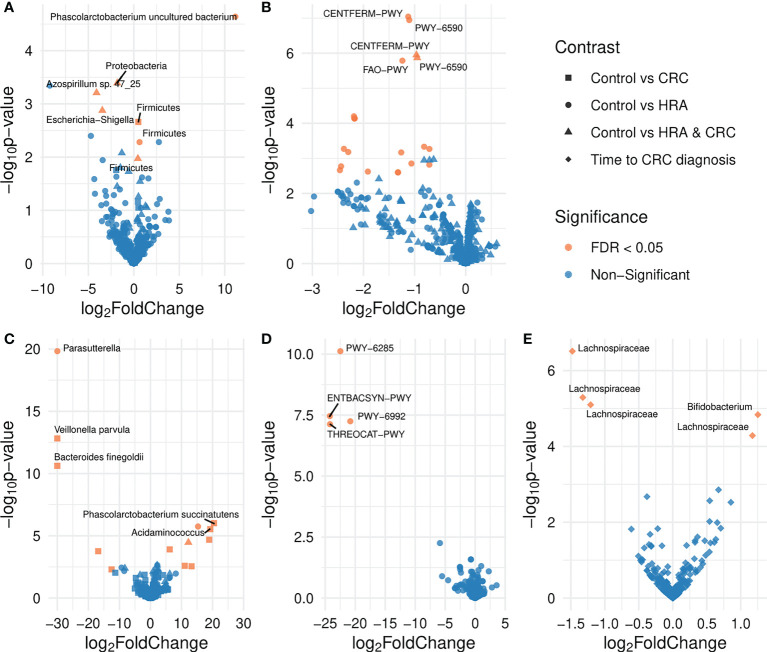
Volcano plots showing differences in the abundance of taxa and pathways between groups. FDR-significant differentially abundant taxa or pathways are highlighted in red. Group comparisons are indicated by different shapes where the control group or a shorter time to diagnosis is considered the reference. Differential abundance was analyzed for **(A)** amplicon sequence variants from 16S sequencing data, **(B)** MetaCyc pathways derived from 16S sequencing data, **(C)** species abundance based on metagenome shotgun sequencing, **(D)** MetaCyc pathways based on metagenome shotgun sequencing, and **(E)** amplicon sequence variants from 16S sequencing data for the 28 participants who received a CRC diagnosis.

For the 16S data, the ASV *Phascolarctobacterium uncultured bacterium* and the phylum *Firmicutes* were significantly more abundant in CRC than controls (FDR *p* < 0.05, **Table 2**; **Figure 5A**). Similarly, in the metagenome data, *Phascolarctobacterium succinatutens* was significantly more abundant in CRC. For the metagenome data, in total, nine species were differentially abundant (FDR *p* < 0.05, **Table 2**; **Figure 5C**). Five of these were significantly higher in CRC compared with controls, whereas four were significantly lower. The genus *Acidaminococcus* was significantly higher in CRC. Four pathways were significantly lower in CRC compared with controls (FDR *p* < 0.05, **Table 2**; **Figure 5D**).

**Table 2:** Differential abundance analyses of taxa and pathways between CRC, HRA, and healthy controls.

Contrast	baseMean	log2FoldChange	padj	Taxa/pathway
ASV taxonomy, *n* = 143				
Control *vs*. CRC	41.37	11.16	0.0072	*Phascolarctobacterium uncultured bacterium*
Control *vs*. CRC	61,410.72	0.63	0.0366	*Firmicutes*
Control *vs*. HRA	33.60	−4.10	0.0464	*Azospirillum* sp. *47_25*
Control *vs*. HRA	303.86	−3.47	0.0498	*Escherichia-Shigella*
Control *vs*. HRA	662.44	−1.83	0.0028	*Proteobacteria*
Control *vs*. HRA	61,410.72	0.44	0.0374	*Firmicutes*
Control *vs*. HRA and CRC	61,410.72	0.50	0.0131	*Firmicutes*
ASV pathways, *n* = 143				
Control *vs*. HRA	181.995	−1.125	1.80E−05	CENTFERM-PWY: pyruvate fermentation to butanoate
Control *vs*. HRA	135.426	−1.240	0.0002	FAO-PWY: fatty acid β-oxidation I
Control *vs*. HRA	48.787	−1.325	0.0397	GALACTARDEG-PWY: D-galactarate degradation I
Control *vs*. HRA	48.787	−1.325	0.0397	GLUCARGALACTSUPER-PWY: superpathway of D-glucarate and D-galactarate degradation
Control *vs*. HRA	45.812	−2.299	0.0165	GLYCOL-GLYOXDEG-PWY: superpathway of glycol metabolism and degradation
Control *vs*. HRA	60.372	−2.383	0.0155	GLYOXYLATE-BYPASS: glyoxylate cycle
Control *vs*. HRA	179.086	−0.713	0.0318	HEMESYN2-PWY: heme biosynthesis II (anaerobic)
Control *vs*. HRA	94.118	−0.814	0.0155	PWY-5177: glutaryl-CoA degradation
Control *vs*. HRA	25.662	−2.439	0.0333	PWY-5747: 2-methylcitrate cycle II
Control *vs*. HRA	58.730	−2.176	0.0029	PWY-5855: ubiquinol-7 biosynthesis (prokaryotic)
Control *vs*. HRA	58.730	−2.176	0.0029	PWY-5856: ubiquinol-9 biosynthesis (prokaryotic)
Control *vs*. HRA	58.730	−2.176	0.0029	PWY-5857: ubiquinol-10 biosynthesis (prokaryotic)
Control *vs*. HRA	29.454	−1.915	0.0397	PWY-5920: superpathway of heme biosynthesis from glycine
Control *vs*. HRA	230.973	−1.105	1.80E-05	PWY-6590: superpathway of *Clostridium acetobutylicum* acidogenic fermentation
Control *vs*. HRA	58.730	−2.176	0.0029	PWY-6708: ubiquinol-8 biosynthesis (prokaryotic)
Control *vs*. HRA	60.697	−1.262	0.0165	PWY0-1415: superpathway of heme biosynthesis from uroporphyrinogen-III
Control *vs*. HRA	132.568	−1.063	0.0317	PWY0-1533: methylphosphonate degradation I
Control *vs*. HRA	25.410	−2.461	0.0397	PWY0-42: 2-methylcitrate cycle I
Control *vs*. HRA	468.521	−0.711	0.0155	REDCITCYC: TCA cycle VIII (*Helicobacter*)
Control *vs*. HRA	56.227	−2.187	0.0029	UBISYN-PWY: superpathway of ubiquinol-8 biosynthesis (prokaryotic)
Control *vs*. HRA and CRC	181.995	−0.965	0.0002	CENTFERM-PWY: pyruvate fermentation to butanoate
Control *vs*. HRA and CRC	230.973	−0.949	0.0002	PWY-6590: superpathway of *Clostridium acetobutylicum* acidogenic fermentation
Metagenome taxonomy, *n* = 46				
Control *vs*. CRC	3,436.272	−30	1.07E−09	*Bacteroides finegoldii*
Control *vs*. CRC	1,543.539	11.06101	3.12E−02	*Lactobacillus rogosae*
Control *vs*. CRC	3,173.952	−12.5276	4.94E−02	*Monoglobus pectinilyticus*
Control *vs*. CRC	80,657.51	6.196732	2.21E−03	*Coprococcus eutactus*
Control *vs*. CRC	4,742.03	−16.8273	2.57E−03	*Roseburia* sp. *CAG:303*
Control *vs*. CRC	1,160.284	13.24881	3.12E−02	*Firmicutes bacterium CAG:95*
Control *vs*. CRC	3,457.533	18.9179	4.59E−04	*Acidaminococcus intestine*
Control *vs*. CRC	19,999.34	20.45545	2.88E−05	*Phascolarctobacterium succinatutens*
Control *vs*. CRC	2,400.172	−30	1.35E−11	*Veillonella parvula*
Control *vs*. CRC	3,264.922	19.253	0.0001	*Acidaminococcus*
Control *vs*. HRA	511.8308	15.320	0.0002	*Clostridium saccharolyticum*
Control *vs*. HRA	1,584.142	−30.000	7.13E−19	*Parasutterella*
Control *vs*. HRA and CRC	511.8308	12.21184	0.0031	*Clostridium saccharolyticum*
Metagenome pathways, *n* = 46				
Control *vs*. CRC	13.053	−24.231	5.64E−06	ENTBACSYN-PWY: enterobactin biosynthesis
Control *vs*. CRC	20.312	−22.480	2.46E−08	PWY-6285: superpathway of fatty acid biosynthesis (*E. coli*)
Control *vs*. CRC	5.721	−20.822	6.05E−06	PWY-6992: 1.5-anhydrofructose degradation
Control *vs*. CRC	3.859	−24.204	6.05E−06	THREOCAT-PWY: superpathway of L-threonine metabolism
ASV taxonomy: time to CRC diagnosis, *n* = 28				
Time	18.42315	1.251612	0.0008	*Bifidobacterium*
Time	19.47221	−1.20951	0.0006	*Lachnospiraceae*
Time	11.94936	−1.47904	0.0001	*Lachnospiraceae*
Time	7.238016	−1.32495	0.0006	*Lachnospiraceae*
Time	13.97661	1.171725	0.0023	*Lachnospiraceae*

Log2FoldChange indicates the magnitude and direction of difference in abundance. Analyses were adjusted for sex and screening center. p-values were adjusted using FDR.

#### 3.3.2 HRA *vs*. control

For 16S data, the genera *Azospirillum* sp. *47_25* and *Escherichia-Shigella* were lower in HRA compared with controls (FDR *p* < 0.05, **Table 2**; **Figure 5A**). The phyla *Proteobacteria* and *Firmicutes* were lower and higher in HRA compared with controls, respectively. The direction of differences for these phyla was similar in the metagenome data, though not significant. Twenty pathways were lower in HRA based on 16S data. Of these, three pathways were related to heme biosynthesis: HEMESYN2-PWY [heme biosynthesis II (anaerobic)], PWY-5920 (superpathway of heme biosynthesis from glycine), and PWY0-1415 (superpathway of heme biosynthesis from uroporphyrinogen-III) (FDR *p* < 0.05, **Table 2**; **Figure 5B**). The direction was similar for PWY0-1415 in the metagenome data. We also observed differences in REDCITCYC [tricarboxylic acid (TCA) cycle VIII (*Helicobacter*)] and the closely related pathways PWY0-42 (methylcitrate cycle I), PWY-5747 (methylcitrate cycle II), and GLYOXYLATE-BYPASS (glyoxylate cycle). For metagenome data, the species *Clostridium saccharolyticum* was significantly higher and the genus *Parasutterella* was significantly lower in HRA compared with controls (FDR *p* < 0.05, **Table 2**; **Figure 5C**).

#### 3.3.3 HRA and CRC *vs*. control

For 16S, when considering HRA and CRC as one group and comparing it with controls, the phylum *Firmicutes* was significantly higher in HRA/CRC (FDR *p* < 0.05, **Table 2**; **Figure 5A**). The same non-significant trend was observed in the metagenome data. The pathways CENTFERM-PWY (pyruvate fermentation to butanoate) and PWY-6590 (superpathway of *Clostridium acetobutylicum* acidogenic fermentation) were lower in HRA/CRC (FDR *p* < 0.05, **Table 2**; **Figure 5B**). For metagenome data, the species *C. saccharolyticum* was significantly more abundant in the HRA/CRC group (FDR *p* < 0.05, **Table 2**; **Figure 5C**).

#### 3.3.4 Time to diagnosis

Assessing the CRC group only, those with a longer interval between sample collection and diagnosis had a higher abundance of one genus, *Bifidobacterium*, and one ASV within the *Lachnospiraceae* family. Additionally, three ASVs within *Lachnospiraceae* were lower in those with a long time to diagnosis (FDR *p* < 0.05, **Figure 5E**; **Table 2**).

## 4 Discussion

Using both 16S rRNA and metagenome sequencing data, we analyzed the microbial differences between CRC, HRA, and healthy controls of the 144 screening attendees with long-term follow-up data. *Phascolarctobacterium* spp. were more abundant in the CRC compared with controls and four ASVs belonging to the *Lachnospiraceae* family, and *Bifidobacterium* was associated with time to CRC diagnosis. Several heme biosynthesis pathways were less abundant in HRA. We did not observe compositional differences between CRC, HRA, and healthy controls and identified no correlation between richness and time to diagnosis in the CRC group.

We identified *Phascolarctobacterium uncultured bacterium* and *P. succinatutens* in the 16S and metagenome data, respectively, as being significantly higher in CRC compared with healthy controls. These annotations likely represent the same species. Three studies have reported similar findings (40–42). Interestingly, Yachida et al. found an elevation in *P. succinatutens* in the early stages of CRC, from polypoid adenomas to stage 1 CRC. *Phascolarctobacterium succinatutens* is broadly distributed in the GI tract and converts succinate into propionate (42). The strain can likely not ferment any other short-chain fatty acids or carbohydrates (43). Succinate is a TCA cycle intermediate and is produced both by the host and the microbiota, including the CRC-associated bacteria *B. fragilis* and *F. nucleatum.* Increased succinate in the colon has been linked to gut inflammation and disease, while increased propionate is thought to be anti-inflammatory (44, 45). Succinate is proposed to mediate cross-talk as a signaling metabolite that acts as a positive regulator of intestinal gluconeogenesis (45, 46) and thermogenesis (47). We also report several pathways related to the TCA cycle to be lower in HRA compared with controls. Vogtmann et al. found this pathway to be increased in cancer (48).

Three pathways related to heme biosynthesis were significantly lower in the HRA group compared with controls. While heme uptake, biosynthesis, and export in bacteria are not fully understood (49, 50), bleeding tumors release heme into the gut lumen. This might create a niche for heme-scavenging bacteria that could outcompete those who rely on heme biosynthesis.

*Bifidobacterium* and four ASVs belonging to the *Lachnospiraceae* family were associated with time to diagnosis. *Bifidobacterium* is a lactic acid-producing bacteria, aiding in colonocyte renewal and inhibiting the growth of pathogens. Two studies found *Bifidobacterium* to be lower in individuals with lesions compared with controls (3, 51). This is in line with our findings that lower levels are associated with a shorter time to diagnosis. We observed different members of the *Lachnospiraceae* family showing diverging associations with time to diagnosis. This family was found to be enriched in controls compared with patients with lesions (52). Some members of the *Lachnospiraceae* family can produce the short-chain fatty acid butyrate (53). Butyrate aids in the cell renewal of colonocytes, serves as a carbon source for the TCA cycle, and has anti-inflammatory and antitumorigenic properties (54, 55).

In contrast to several cross-sectional studies including established CRC cases (10, 11, 56), we did not observe any associations of bacteria including *F. nucleatum*, *E. coli*, and *B. fragilis* with CRC status when assessing mostly prediagnostic cases. These findings could indicate that shifts in the abundance of these bacteria might be late events in colorectal carcinogenesis.

We found no difference in diversity or composition between CRC, HRA, and controls. Results from similar studies seem to be conflicting, both for diversity and composition analyses (11, 56–59). Smaller differences in the microbiome of adenomas and healthy controls have been observed than those observed between cancers and healthy controls (3, 11). Unlike previous studies in the field, many of our samples were collected from asymptomatic subjects, years before the diagnosis of cancer. While our results indicate no overall difference in diversity or composition, it is possible that we have been underpowered or that factors related to study design and technical challenges have led us to miss any small differences in these ecological measures.

This study has some other noteworthy limitations. Firstly, our samples were stored for 17 years and could possibly be degraded. We do know that these samples have a maximum of one freeze–thaw cycle (22), but they were stored without a stabilizing agent, which could to some extent influence the composition of fecal samples (60, 61). Furthermore, we lack information on important confounding factors, such as diet, lifestyle factors, body mass index, and antibiotic use affecting microbiome composition (54, 62). Confounding by these factors may have introduced false-positive associations, and although the large effect sizes observed in many cases could suggest a causal relationship, ultimately, our findings will need to be validated in larger studies controlling for lifestyle factors. Lastly, we observed a high abundance of the phylum *Firmicutes* in our 16S data, but a similar composition was not observed for the metagenome data. This is likely due to the choice of primers, where for marker gene studies, certain primers favor the amplification of specific taxa (63). Still, this did likely not affect the differential abundance analyses, as the bias was uniform across samples.

## 5 Conclusions

The present study is, to the best of our knowledge, the first to examine gut microbiome samples collected several years prior to CRC diagnosis. We did not find any differences between the diversity and composition of the gut microbiome and the presence of CRC, HRA, and controls. However, analyses identified several taxa and pathways that were differentially abundant. Our study found that the succinate-metabolizing, associated with inflammation, *P. succinatutens* was more prevalent in individuals diagnosed with CRC than in healthy controls, identified using both 16S and metagenome data. In this population-based screening setting, we also show that CRC-associated taxa are identifiable years prior to diagnosis of CRC, including *Bifidobacterium* and *Lachnospiraceae*, which were associated with time to diagnosis.

## Data availability statement

“Due to the sensitive nature of the data derived from human subjects, including personal health information, our institution has a strict policy on data sharing to ensure the privacy and confidentiality of our participants. Data and/or biological material from this project must comply with the General Data Protection Regulation (GDPR). This means that the processing must have approval from the Regional Committee for Medical Research in Norway (REC). Furthermore, the processing needs legal basis according to GDPR Article 6 and 9 and the need for a Data Protection Impact Assessment (DPIA) according to GDPR article 35 must be considered. Disclosure of information and/or biological material to countries outside the EU requires that the conditions in GDPR are met. Although we are unable to deposit the raw data in a public database to protect the privacy of our participants, we are willing to collaborate with researchers who wish to use our data by providing summary statistics and analyses upon request. Requests for data access can be directed to Trine B Rounge, trinro@uio.no.”

## Ethics statement

The studies involving human participants were reviewed and approved by the Regional Committees for Medical and Health Research Ethics in South-Eastern Norway. The patients/participants provided their written informed consent to participate in this study. Written informed consent was obtained from the individual(s) for the publication of any potentially identifiable images or data included in this article.

## Author contributions

TBR and GH were responsible for the conception and design of the study. CBJ and EB analyzed the data. EV and VB performed the sample preparation and lab work. CBJ, EB, GH, PB and TBR interpreted the data. CBJ, EB, and TBR drafted the manuscript. All authors have revised, commented on, and approved the final submitted manuscript.
