# Supplementary material for: Long-term follow-up of colorectal cancer screening attendees identifies differences in Phascolarctobacterium spp. using 16S rRNA and metagenome sequencing
Source: Front Oncol. 2023 Apr 27;13:1183039. doi: 10.3389/fonc.2023.1183039 (PMC10172651; doi:10.3389/fonc.2023.1183039)
Supplement: Supplementary file 1 [file DataSheet_1.docx]

Supplementary information: Bucher-Johannessen et al, Long term follow-up of colorectal cancer screening attendees identifies differences in *Phascolarctobacterium spp*. using 16S rRNA and metagenome sequencing.


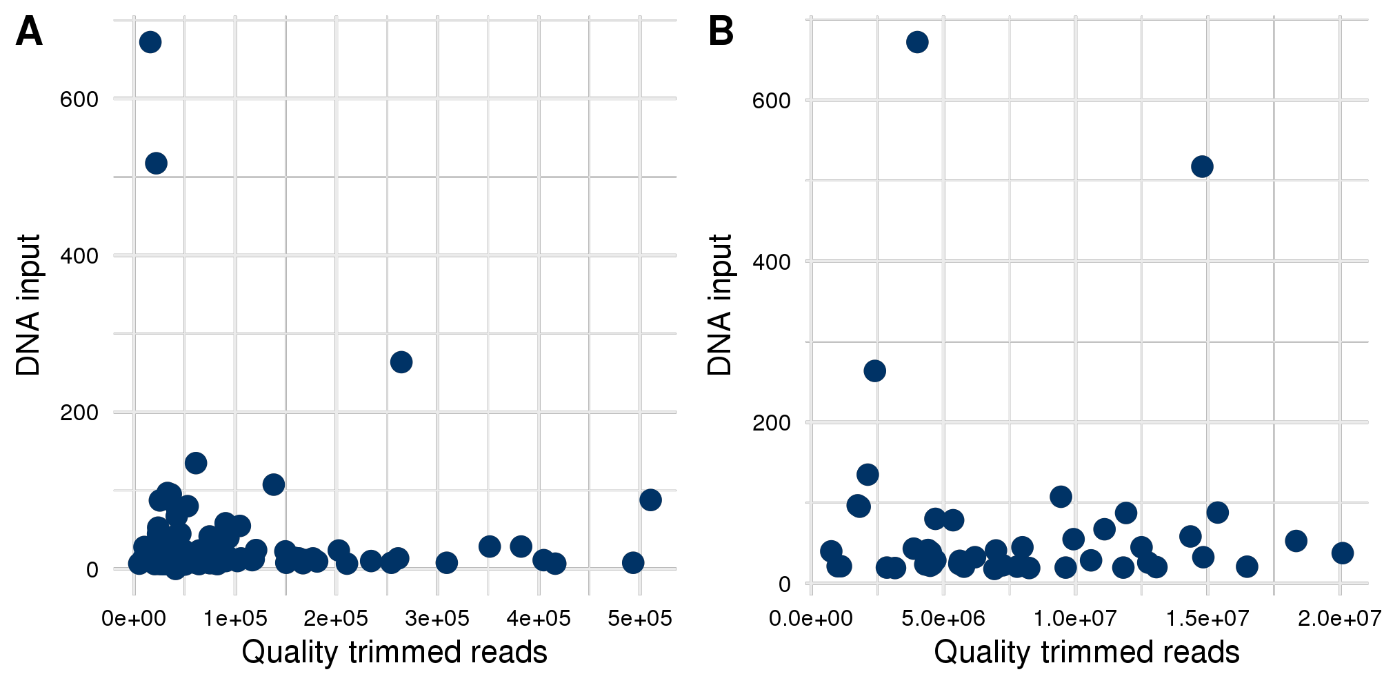


Figure S1 Quality trimmed reads plotted against DNA concentration after DNA isolation for the A) 16S rRNA data B) Metagenome data


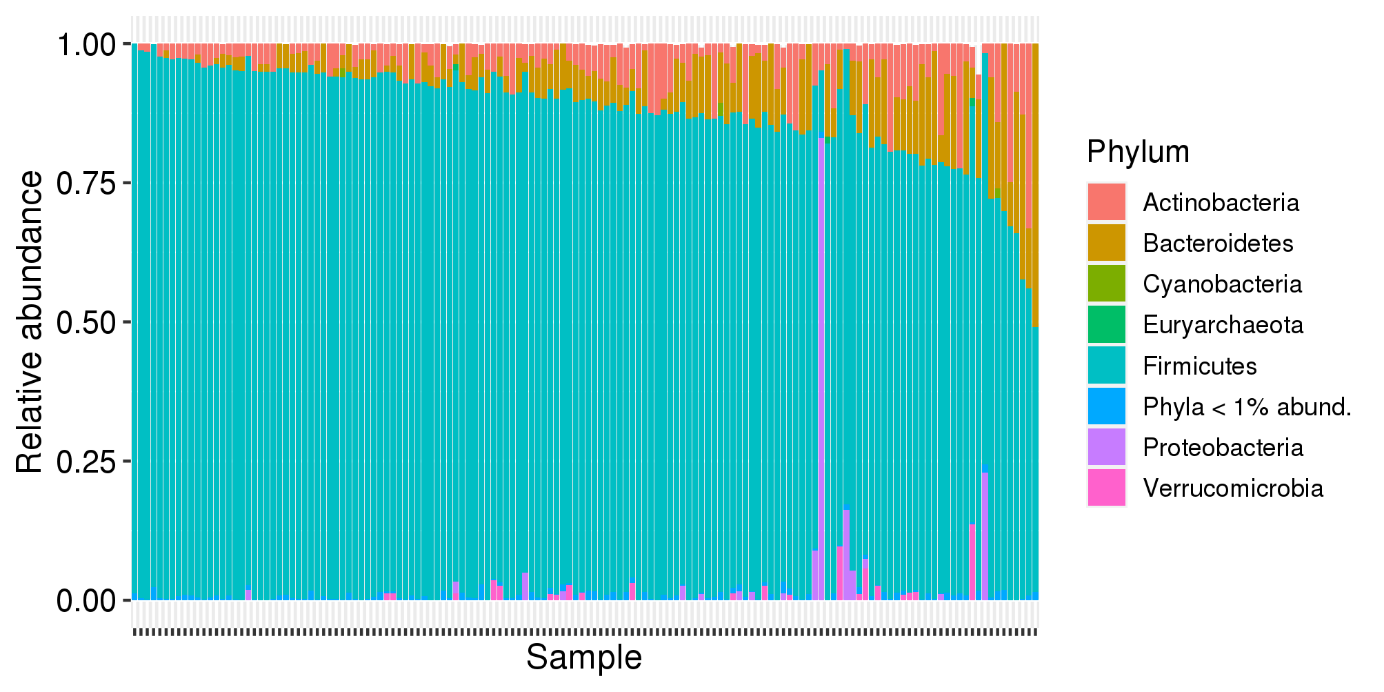


Figure S2 Relative abundance plot on phylum level for all samples from the 16S rRNA data.
